# Herbicidal effects of wood vinegar on nitrophilous plant communities

**DOI:** 10.1002/fes3.253

**Published:** 2020-10-12

**Authors:** Juan Luis Aguirre, Juan Baena, María Teresa Martín, Sergio González, José Luis Manjón, Manuel Peinado

**Affiliations:** ^1^ Cátedra de Medio Ambiente Facultad de Ciencias de la Vida Universidad de Alcalá Madrid Spain; ^2^ Environment and Bioproducts Group Facultad de Ciencias de la Vida Universidad de Alcalá Madrid Spain; ^3^ Instituto Franklin de Estudios Norteamericanos Universidad de Alcalá Madrid Spain; ^4^ Departamento de Ciencias de la vida Facultad de Ciencias de la Vida Universidad de Alcalá Madrid Spain

**Keywords:** biomass pyrolysis, glyphosate, herbicide, nitrophilous vegetation, weed control, wood vinegar

## Abstract

In Europe, and many parts of the world, the number and variety of animal species on farmland is in marked decline. There is a need to search for alternatives that are safe for the environmental and are effective in controlling weeds. Wood vinegar from biomass pyrolysis may be an alternative for herb control. In this study, Wood vinegar (WV) pH, moisture content, and composition were analyzed, with subsequent assessment of the effects of WV on nitrophilous plant communities under natural conditions. The following three treatments were used: WV dissolved in water to form 25 vol% and 50 vol% dilutions and undiluted WV (100 vol%). The results showed a greater than 70% decrease in biomass at 7 days after WV application in all treatments. At the end of the sampling period (day 42), the plots treated with WV had four‐times less biomass than the controls. No significant differences were observed among different treatments, thus indicating that a 25% dilution may suffice for use as an herbicide. However, this concentration also produced the highest variability in results. The area cleared by the affected species was colonized by perennial species. At the end of the sampling, 80% of the area of the treated plots was occupied by perennial species, whereas this percentage was 30% in control plots. Electron micrographs showed that the epidermis of the treated plants was severely affected within a few hours of the treatment, particularly of the stomatal cells. The most affected species were those with smooth leaves without protective structures and those with lighter stems and leaves. The good herbicidal performance of WV notwithstanding, regulations must be clarified for its use as an herbicide.

## Graphical abstract

Treatment with WV helps to control the development of annual plants in ruderal communities. WV is a useful compound for some herb‐control treatments, effectively controlling biomass development, and replacing systemic herbicides

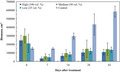

## 1 INTRODUCTION

The adverse health effects associated with glyphosate, the most widely used herbicide worldwide, is the focus of intense regulatory debate. With studies opposed, some of them confirmed the effects (Zhang et al., 2019) and other studies in other way (Andreotti et al., 2018; Crump, 2020), glyphosate was classified as “probably carcinogenic” for humans in March 2015 by the International Agency for Research on Cancer (IARC, 2015). Actually, many countries, regions, and municipalities have banned or are strictly regulating its use (http://sustainablepulse.com). Therefore, there is an urgent need to search for alternatives that are safe for the environmental and human health and are effective in controlling weeds.

Wood vinegar (WV), or pyroligneous acid, is the aqueous fraction resulting from wood pyrolysis, which has been used for decades as a food additive (Guillén & Manzanos, 2005). This bioproduct contains a wide variety of oxygenated organic compounds, such as acids, alcohols, phenols, esters, and sugars, with acetic acid as its primary compound (Fagernäs et al., 2012; Martín et al., 2017; Mathew & Zakaria, 2015; Wang et al., 2010).

Furthermore, pyrolysis of lignocellulosic waste is a useful tool to assess lignocellulosic residues while avoiding environmental problems derived from their accumulation in the environment (Aguirre et al., 2020). In addition to WV, other by‐products such as biochar, syngas, bio‐bitumen, and bio‐oil with various applications are generated during the pyrolysis of lignocellulosic waste (Hunter et al., 2017; Martín et al., 2017; Zhang et al., 2007).

Numerous studies on WV have been conducted (Tiilikkala et al., 2010), to analyze its fungicidal, antioxidant, fertilizer, and antiviral properties, and even as an animal fodder option (Grewal et al., 2018; Pan et al., 2017; Rahmat et al., 2014; Ratnani & Widiyanto, 2018). However, only a few studies on its effect as an herbicide under natural conditions have been published, and most of those studies were conducted in greenhouses, growth chambers, or single‐species plots (Ruuttunen, 2007; Salonen et al., 2008; Tworkoski, 2002).

Research studies on the herbicidal effects of WV have evaluated the effects of WV treatments a few days after its application. However, in nitrophilous plant communities are subjected to dynamic changes over time. In Mediterranean climate zones, the first plants emerge during the early spring. The communities grow until the arrival of summer when drought afflicts these areas (early summer). During this season, several species compete with each other to survive. Therefore, this succession must be analyzed to understand how an herbicide would work under natural conditions.

Therophytes are annual plants that complete their cycle within a few weeks. Thus, several waves of emergence of different species usually occur, with the disappearance of the most ephemeral, early‐flowering species and the establishment of late‐flowering species (Bartolomé et al., 1988; Loidi, 2017). These communities consist of opportunistic plants that take advantage of cleared land, high concentrations of anthropogenic nitrates, and abundant organic matter available thanks to their growth strategy. These plants are ruderals (Grime, 1974), based on the *r*‐selection model (MacArthur & Wilson, 1967), whose life cycle is very fast and short. With the first spring rains, the abundant seed bank of the soil germinates and plants grow rapidly, producing long leaves and stems to fight for access to light, flowering within a few days.

This ruderal demographic strategy has helped these plants to increase their area of distribution and have benefited from human expansion. For this reason, most species are cosmopolitan and form perfectly structured communities in all temperate regions of the world, either as dominant species or associated with local or regional endemic species that utilize the same strategy (Mucina et al., 2016).

The results from various types of WV treatments performed in a growth chamber using different nitrophilous plant species were evaluated in a recent publication (Aguirre et al., 2020). The authors concluded that concentrations from 25 to 100 vol% WV were effective in controlling annual herb species under laboratory conditions. The objective of the present study is to assess the effect of WV under natural conditions using the concentrations effective under controlled growth chamber conditions and to observe the evolution of nitrophilous communities herbs treated with WV. In addition, an electron microscopy was performed to observe the cellular damage inflicted by WV on the ruderal plant structures to determine the mechanism of action of this phytosanitary product.

## 2 MATERIAL AND METHODS

### 2.1 Wood vinegar characterization

WV, derived from pine pyrolysis, was procured from Neoliquid Advanced Biofuels and Biochemicals S.L. (Guadalajara, Spain). WV was characterized using different methods. The pH was determined using a 692 pH/Ion Meter (Metrohm) equipped with a Solitrode glass electrode with a temperature probe Pt1000 (Metrohm). The water content was calculated by Karl Fischer titration using a Metrohm oven sample processor. Gas chromatography was performed using the conditions described in previous studies (Aguirre et al., 2020; Martín et al., 2017). An Agilent 7,890 A gas chromatograph coupled with a 5,977 A single quadrupole mass spectrometer (Agilent Technologies) and a Phenomenex ZB‐624 column (30 m; 0.25 mm i.d.; 0.25 μm film thickness, 6% cyanopropylphenyl/94% dimethylpolysiloxane) was used for this purpose. The quantification was performed using the internal standard method, by constructing a calibration curve for each of the following compounds: acetic acid (40–515 ppm), hydroxyacetone (40–515 ppm), hidroxyacetaldehyde (50–960 ppm), furfural (10–110 ppm), and levoglucosan (10–200 ppm). A constant concentration of internal standards (IS) 1‐octanol (830 ppm) and cresol (115 ppm) was added at each point on the calibration lines. Quantification was performed based on the areas determined by extracting the quantifying ion from each compound, in both standards and samples.

### 2.2 Field experiments

The field experiments were conducted using the facilities of the Royal Botanic Gardens, University of Alcalá (*Universidad de Alcalá* – UAH), with communities of natural weeds. In addition, the soil in the field was rich in seeds of ruderal communities of the area and had not been previously treated with herbicides (Bartolomé & Díaz, 2006). The experiment was performed during the natural sprouting season of these communities, which usually spans from April to early June in the Mediterranean climate zones of the northern hemisphere. The WV was applied only once in April through a standard product application backpack.

The method used in the study was nondestructive and had no adverse effects on the environment where the experiment was conducted to allow observation of the natural temporal evolution of the vegetation. All plant species of the plots were identified as *Flora Ibérica* (Iberian Flora) (Castroviejo, 1986). Subsequently, the partial cover of each plant species in each plot was estimated by using sigmatist phytosociological methods and expressed as the percentage of area occupied by each species in the plot (Peinado et al., 2008). The sum of plant covers can surpass 100% because these communities are composed of wide variety of plants ranging from creeping plants to large, erect plants, which can exceed a meter in height. In addition, the average plant height was measured in the plot. Since the plot had plants of different heights, the percentage of area covered by each height was measured at several points, thus calculating the average plant height. A value indicative of the total biomass volume in each plot was calculated by multiplying the plant cover by the average plant height. The first two samplings were performed at 24 and 48 hr and were repeated at 7, 14, 28, and 42 days, after spring ended and the absence of rain stopped further development of these communities. The biomass was used to evaluate the combined results of the plots and the plant cover of each species or group of species.

Scanning electron microscopy (*SEM*) was used to observe foliar damage. Leaves treated with WV were cut in small pieces (5–6 × 4 mm), put into perforated Beem capsules (size 3), and fixed in 6% glutaraldehyde at 4°C for 24 hr or longer. Then, the Beem capsule was drained on a filter paper (1 min) and transferred to 100% acetone for at least 48 hr. This was followed by critical point drying and sputtering with gold–palladium. *SEM* micrographs were obtained at the University of Alcalá (Madrid) using a Zeiss DSM–950 device.

For the sampling design, 4 types of treatments were applied in 1 × 1.5 m plots. Treatments with 25 vol% WV (low group), 50 vol% WV (medium group), and 100 vol% WV (high group) were performed in four plots for each group, for a total of 12 plots. Two control plots received no treatment (control group). The application volume was 0.8 L/m^2^ in 8 plots and 1.6 L/m^2^ in 4 plots, two at 25% WV and two at 50% WV. No irrigation, other than the natural rainfall in the area, or any other type of treatment was performed in the plots.

ANOVA was used, and when the differences between groups were significant, was used Fisher's test The program used for all statistical tests was Statplus 7.1. (AnalystSoft Inc., Walnut, CA, USA).

## 3 RESULTS

### 3.1 Wood vinegar composition

The WV prepared from pine biomass primarily consisted of water (84.20 wt%) derived from the thermal decomposition of hemicellulose (Cai et al., 2019; Demirbaş, 2002; Martín et al., 2017) and from the natural moisture of the pine chips. The other 15.80 wt% consisted of a mixture of water‐soluble organic compounds, as listed in Table 1.

**Table 1:** Wood vinegar composition determined by GC/MS

Compound	Tr (min)	Formula	Area	%area (%wt)
Acids				
Acetic acid	6.96	C_2_H_4_O_2_	27,822,047.9	18.91 (4.34)
Propanoic acid	8.64	C_3_H_6_O_2_	3,376,220.2	2.30
Butanoic acid	9.80	C_4_H_8_O_2_	568,824.7	0.39
Acetic acid, (acetyloxy)‐	9.28	C_4_H_6_O_4_	1,740,139.1	1.18
Esters				
Ethyl Acetate	5.46	C_4_H_8_O_2_	633,817.1	0.43
2‐Propanone, 1‐(acetyloxy)‐	10.60	C_5_H_8_O_3_	1,621,314.6	1.10
2‐Butanone, 1‐(acetyloxy)‐	11.56	C_6_H_10_O_3_	582,796.5	0.40
Butyrolactone	11.67	C_4_H_6_O_2_	1,523,954.7	1.04
9‐Tetradecen‐1‐ol, acetate, (E)‐	12.49	C_16_H_30_O_2_	588,287.5	0.40
2‐Hydroxy‐gamma‐butyrolactone	12.72	C_4_H_6_O_3_	1,224,031.2	0.83
Aldehydes				
Acetaldehyde, hydroxy‐	5.92	C_2_H_4_O_2_	4,832,663.8	3.29 (2.75)
Succindialdehyde	9.76	C_4_H_6_O_2_	342,429.4	0.23
Ketones				
Acetona	3.09	C_3_H_6_O	1,712,183.5	1.16
2‐Propanone, 1‐hydroxy‐	7.73	C_3_H_6_O_2_	20,346,086.4	13.83 (2.21)
Acetoin	8.49	C_4_H_8_O_2_	691,341.2	0.47
1‐Hydroxy‐2‐butanone	9.38	C_4_H_8_O_2_	3,100,889.4	2.11
2‐Cyclopenten‐1‐one	10.28	C_5_H_6_O	1,739,728.7	1.18
5,9‐Dodecadien‐2‐one, 6,10‐dimethyl‐, (E,E))‐	10.69	C_14_H_24_O	475,430.8	0.32
2‐Cyclopenten‐1‐one, 2‐methyl‐	11.00	C_6_H_8_O	1,000,657.6	0.68
2‐Cyclopenten‐1‐one, 2‐hydroxy‐	11.36	C_5_H_6_O_2_	1,059,437.0	0.72
Alcohols				
1,2‐Ethanediol	8.88	C_2_H_6_O_2_	1,926,727.7	1.31
1,3‐Propanediol	9.14	C_3_H_8_O_2_	402,176.7	0.27
1,2,3‐Cyclopentanetriol	11.21	C_5_H_10_O_3_	506,637.5	0.34
2‐Cyclohexen‐1‐ol	11.47	C_6_H_10_O	590,309.5	0.40
Cyclopentanol	13.49	C_5_H_10_O	2,027,616.8	1.38
Phenols				
Phenol	12.28	C_6_H_6_O	5,662,039.6	3.85
Phenol, 2,5‐dimethyl‐	13.53	C_8_H_10_O	1,341,965.7	0.91
Catechol	14.71	C_6_H_6_O_2_	4,764,242.4	3.24
Carbohydrates				
D‐Mannose	17.29	C_6_H_12_O_6_	3,335,344.9	2.27
D‐Allose	18.21	C_6_H_12_O_6_	3,834,671.9	2.61
Levoglucosan	19.39	C_6_H_10_O_5_	12,204,882.9	8.30 (1.09)
Furans				
2‐Furanol, tetrahydro‐	9.64	C_4_H_8_O_2_	443,860.9	0.30
2‐Furanmethanol, tetrahydro‐	10.03	C_5_H_10_O_2_	367,763.9	0.25
Furfural	10.21	C_5_H_4_O_2_	3,151,605.4	2.14 (0.38)
2‐Furanmethanol	10.54	C_5_H_6_O_2_	1,153,934.0	0.78
3‐(3*H*)‐Furanone	11.71	C_4_H_4_O_2_	2,599,322.7	1.77
Furan, 2,5‐dihydro‐2,5‐dimethyl‐	11.89	C_6_H_10_O	1,396,881.4	0.95
4‐Methyl‐5*H*‐furan‐2‐one	12.24	C_5_H_6_O_2_	342,815.3	0.23
Nitrogenous				
Acetic acid, hydrazide	3.42	C_2_H_6_N_2_O	825,170.7	0.56
Hydrazine, methyl‐	6.81	CH_6_N_2_	411,547.7	0.28
Ethanone, 1‐(1*H*‐pyrazol‐4‐yl)‐	11.08	C_5_H_6_N_2_O	312,413.7	0.21

In total, 42 organic compounds were identified, all of which were oxygenated, accounting for 83.33% of the area of the chromatogram. The other 16.67% of the area of the chromatogram corresponded to coelutes and unidentified compounds.

The percentage of area assessed by GC/MS provides data on the relative content of each compound in the study sample. However, these data are not quantitative, and assessing their exact concentration requires quantification of each compound. In this case, the following five major compounds were quantified: acetic acid, 1‐hydroxy‐2‐propanone, hydroxyacetaldehyde, furfural, and levoglucosan (Table 1). The concentration of acetic acid was 4.34 wt%, while the concentrations of hydroxyacetaldehyde and 1‐hydroxy‐2‐propanone were lower (2.75 and 2.21 wt%, respectively). In turn, the concentration of levoglucosan was 1.09 wt%, while that of furfural was 0.38 wt%. In total, the quantified compounds accounted for 10.77 wt% of WV weight analyzed. The remaining 5.03 wt% of the WV composition corresponded to unquantified identified compounds, co‐evolutions, and unidentified compounds. It should be pointed out that polycyclic aromatic hydrocarbons (PAH) were not detected in the wood vinegar GC/MS analysis.

### 3.2 Effect on ruderal species

Plant cover and initial biomass showed no differences between plots, as indicated by the values of the three treatments and of the two control plots, which were analyzed for both plant cover and biomass. No significant differences were found at the beginning of the treatment (Table 2).

**Table 2:** Coverage and biomass average in each treatment (Average and standard deviation)

Treatment	Group High (100 vol%)	Group Medium (50 vol%)	Group Low (25 vol.%)	Control
Cover (cm^2^)	15,870 ± 2,636	15,030 ± 4,752	17,210 ± 2083	13,875 ± 530
Biomass (cm^3^)	200,100 ± 86,816	263,580 ± 149,099	302,625 ± 100,666	152,250 ± 13,788

Note

There were no significant differences between the groups (ANOVA, *F* = 1.76, *p* = .22 and *F* = 1.36, *p* = .31).

#### 3.2.1 Biomass results

**Figure 1:**
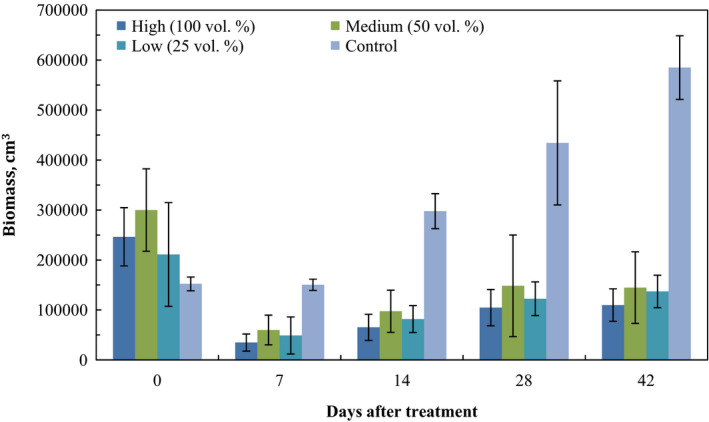
Biomass evolution over time after treatment with different doses of wood vinegar dilutions. There are significant differences between control and treatments at all intervals. No significant differences were observed between treatments

**Figure 2:**
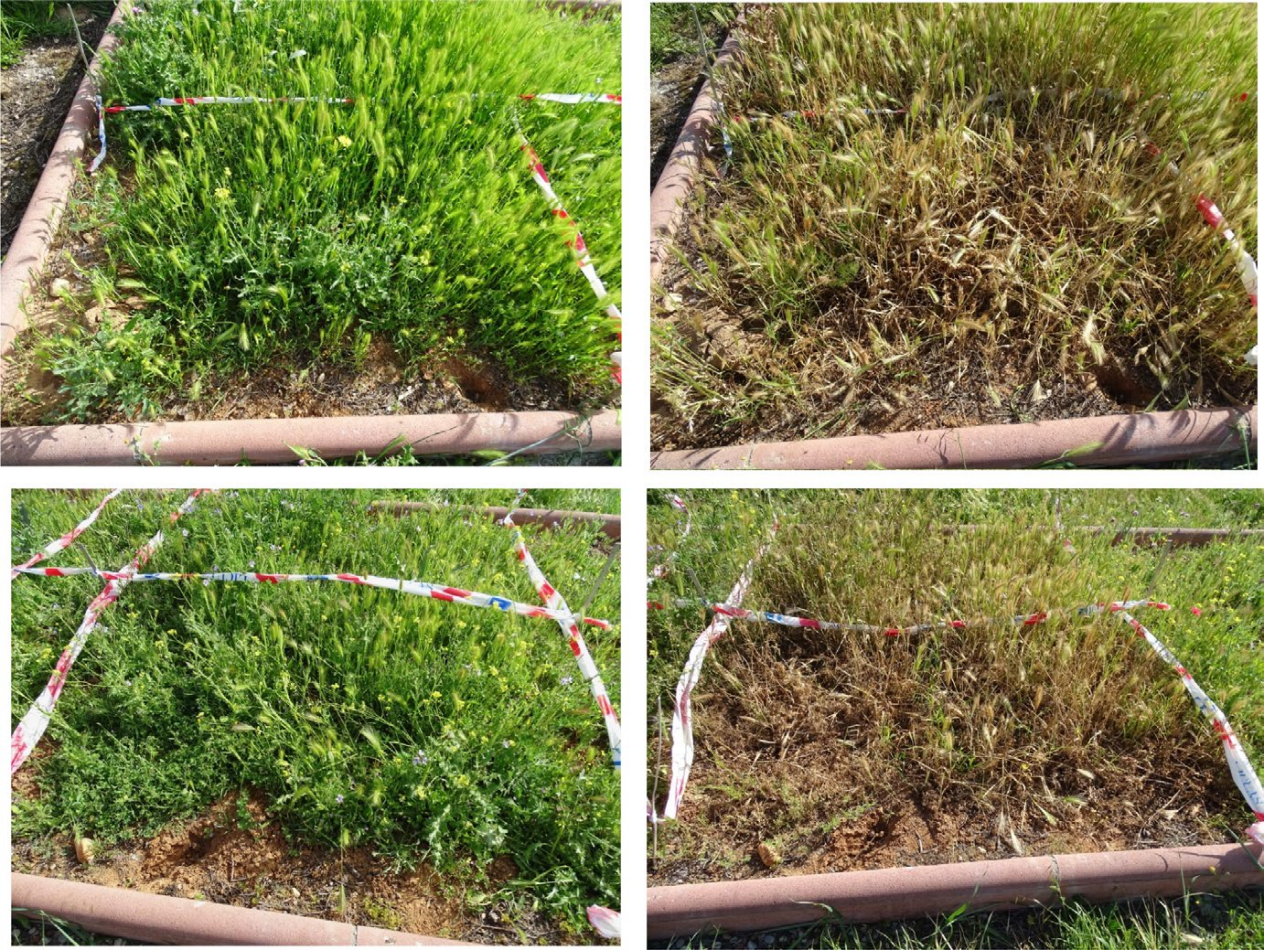
Two different plots with 100 vol% WV treatment, at day 0 (left) and at day 7 (right)

Effects of treatment were visible after 24 hr, with a subsequent biomass reduction of over 75% in all treatments by day 7, while no changes occurred during the first week in the control plots (Table 3). This difference was significant among all groups and the control, but not among the high, medium, and low groups. The evolution between dates indicates that the biomass steadily decreased in the plots until the end of the sampling (Figure 1), albeit increased little by little (significant differences in means between control and treatments on all dates and in all groups; ANOVA, *p* < .001).Figure 2 shows the effects of the treatment with 100% WV after 7 days.

**Table 3:** Percentage of biomass reduction from day 0, at each sampling interval. Fisher LSD *p* < .001, indicating significant differences between groups H, M, and L and the control group, but not between groups (*p* > .05)

Interval	High (100 vol%)	Medium (50 vol%)	Low (25 vol%)	Control
From 0 to 7 days	−84.56% ± 10.29	−81.04% ± 6.51	−78.27% ± 5.39	−1.11% ± 1.57
From 0 to 14 days	−71.45% ± 16.41	−68.78% ± 7.59	−56.09% ± 19.41	+95.33% ± 5.30
From 0 to 28 days	−54.02% ± 25.93	−53.68% ± 25.57	−36.42% ± 16.93	+190.10% ± 107.78
From 0 to 42 days	−53.35% ± 17.48	−54.37% ± 15.04	−15.94% ± 61.32	+283.92% ± 7.03

No significant differences were observed in percentage of biomass recovery among different groups (Fisher LSD *p* > .05 in all cases). The increase in biomass was greater in the first intervals than in the final interval (between days 28 and 42, ANOVA *F* = 9.01, *p* < .001). These differences between intervals were significant in all groups (Fisher LSD, *p* < .05 between intervals) (Table 4).

**Table 4:** Percentage of biomass increase between intervals

Treatment	From day 7 to 14	From day 14 to 28	From day 28 to 42
High (100 vol%)	95.08% ± 26.94	63.61% ± 15.04	9.62% ± 25.07
Medium (50 vol%)	74.26% ± 49.92	42.56% ± 48.12	6.41% ± 22.99
Low (25 vol%)	112.25% ± 104.86	56.68% ± 51.96	24.64% ± 69.91
Control	97.59% ± 8.50	49.33% ± 59.23	42.63% ± 55.42

#### 3.2.2 Plant cover

**Figure 3:**
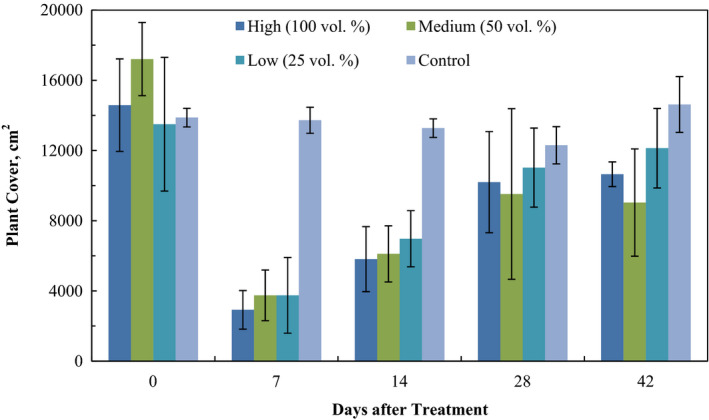
Plant cover evolution over time after treatment with different doses of wood vinegar dilutions. Significant differences were observed between control and treatments at days 7 and 14, but not at days 28 and 42

Plant cover exhibited greater than 75% decrease during the first 7 days, followed by a gradual recovery until day 42 (Table 5). No significant differences were observed among different treatments, but the differences between the control and the treatments were significant (Figure 3).

**Table 5:** Percentage of plant cover reduction from day 0 at each sampling interval

Interval	High	Medium	Low	Control
From 0 to 7 days	−78.84% ± 11.21	−78.69% ± 6.37	−73.25% ± 8.62	−1.10% ± 1.6
From 0 to 14 days	−58.71% ± 17.44	−64.57% ± 7.32	−44.26% ± 23.03	+4.33% ± 0.17
From 0 to 28 days	−26.39% ± 34.30	−44.73% ± 27.86	−15.42% ± 17.81	+11.43% ± 4.26
From 0 to 42 days	−24.63% ± 18.55	−47.64% ± 16.97	−0.44% ± 49.89	+5.70% ± 15.51

Significant differences were observed among groups H, M, and L and the control group at days 7 and 14, but not between treatments (*F* = 45.73, *p* < .01; *F* = 6.82, *p* < .01, Fisher LSD *p* < .01). Moreover, no significant differences were observed among groups on day 28 or day 42 (*F* = 1.11, *p* = .39; *F* = 2.08, *p* = .1).

The increase in plant cover assessed in treatment plots is shown in Table 6.

**Table 6:** Percentage of plant cover increase between different intervals

Treatment	From 7 to 14 days	From 14 to 28 days	From 28 to 42 days
High	102.37% ± 23.41	82.28% ± 40.70	9.70% ± 25.15
Medium	74.26% ± 49.92	50.97% ± 47.54	2.49% ± 24.58
Low	124.75% ± 113.49	63.07% ± 41.18	14.32% ± 38.41
Control	−3.24% ± 1.37	−7.43% ± 4.29	19.91% ± 23.27

The increase in plant cover assessed in treatment plots was higher in the initial intervals, from 7 to 14 days and from 14 to 28 days, than in the final interval (from 28 to 42 days, Table 6). (Fisher LSD *F* = 3.78, *p* < .001; *F* = 2.24, *p* < .05), with no significant differences between the intervals 7/14 and 14/28 (*F* = 1.54, *p* = .13).

#### 3.2.3 Effect of WV amount on biomass unit

The effect of WV amount per unit of biomass is shown in Table 7. Although, as indicated above, no significant differences were observed between plots, the results were analyzed to assess whether there was any correlation between the amount of WV per unit of biomass and the percentage of biomass reduction in each interval. No such relationship was found between the amount of WV applied per unit of biomass and the percentage of biomass reduction, in any interval (Pearson correlation *p* > .05 in all cases).

**Table 7:** Pearson correlation between WV volume used per unit of biomass and biomass reduction by interval

	% reduction 7	% reduction 14	% reduction 28	% reduction 42
Volume/Biomass (mm^3^/cm^3^)	R = 0.11 *p* = .73	R = 0.32 *p* = .30	R = 0.25 *p* = .43	R = 0.27 *p* = .39

### 3.3 Results by vascular plant family

In the combined inventories of the plots, 27 vascular plant species representative of Mediterranean nitrophilous communities were detected; however, only some of these plants had significant plant cover values.

At the beginning of the experiment, the plots presented a floristic composition typical of spring in the Mediterranean region, with a predominance of Poaceae or grasses (*Bromus, Avena,* and *Hordeum*), Asteraceae (*Calendula, Carduus, Crepis,* and *Taraxacum*), Geraniaceae (*Erodium* and *Geranium*), and Brassicaceae (*Sysimbrium* and *Capsella*). Most species were annual and only some were perennial (*Medicago sativa* and *Cynodon dactylon*). A few others, such as *Onopordon* and *Eryngium*, were biennial, that is, they develop a basal rosette of leaves in the first year and a flowery stem in the following year.

Together, these plants are diagnostic species of the *Digitario sanguinalis‐Eragrostietea minoris* class, which clusters grass‐rich anthropogenic vegetation rich in summer‐annual C4 species in the Mediterranean zones of Europe experiencing prolonged periods of summer drought. Within this class, the communities in our study plots are listed in the order *Eragrostietalia* and in the alliance *Diplotaxion erucoidis,* two syntaxa dominated by plants of subcosmopolitan and cosmopolitan distribution (see Mucina et al., 2016 for a more extensive description of those syntaxa).

The plots represent an approximate composition of the plant communities found on roadsides, garden areas, orchards, and ditches throughout the Mediterranean region and large areas of temperate regions throughout the Northern Hemisphere. In some places, the specific species may vary, but the floristic typology of the community is quite similar. The behavior of the different families was analyzed and is discussed here focusing on some representative species.

#### 3.3.1 Poaceae

Effect over the different species of the genus *Bromus* (represented in the plots by *Bromus maximus, B. rubens, B. matritensis*, and *B. tectorum*) was higher than 80% from the first application (Table 8), thus confirming previous results for that genus when assessed in a growth chamber (Aguirre et al., 2020). However, for *Hordeum leporinum*, a grass commonly found in a variety of ruderal Mediterranean communities, the reduction was lower (57%). By the time WV was applied, most of the *Hordeum* population was flowering, and the spike was already developed. This species exhibits early phenology, quickly germinating and growing as soon as the first rains fall. After the treatment, rapid drying with a light yellowing was observed, but the plant remained upright. When analyzing the results by treatment type, no significant differences were found among WV treatments with any of the study species. All results, for all species and dates, significantly differed between the control and the treatment plots (ANOVA *p* < .01 in all cases), except for day 42, when most grasses had already completed their cycle and disappeared (ANOVA *p *> .05).

**Table 8:** Percentage of coverage reduction from day 0, for treatments and control

Species	Initial coverage (cm^2^)	Day 7	Day 14	Day 28	Day 42
*Hordeum leporinum*	3,750 ± 1,318.57	−57%	−56.33%	−76.66%	−91%
Control	150 ± 35.36	0%	+3.33%	+3.33%	−100%
*Bromus sp*.	1,087.5 ± 527.91	−88.50%	−77.01%	−86.20%	−100%
Control	150 ± 35.36	0%	+3.33%	+3.33%	−100%
*Cynodon dactylon*	212.5 ± 259.48	+305.88%	+1,029.41%	+2,105.88%	+2,752.94%
Control	750 ± 50.71	+5.20%	+8.34%	+10.01%	+403.33%

Note

Significant differences between the treatment and the control were found in all intervals (except for day 42) and in all species. No significant differences were found between WV treatments (ANOVA *p* > .05). (Although the results were analyzed for each treatment, the results of the WV treatments have been combined in the tables for further clarity.)

Among the Poaceae, *Cynodon dactylon* is one of the recorded perennial species. At the beginning of the sampling, this species had a low plant cover; however, at the end of the sampling period, *C. dactylon* had the highest plant cover in all treatment plots. Plant cover increased throughout the sampling period, with increase greater than 100% in several weeks. At the end of the treatment, many treatment plots showed a virtually continuous plant cover of this species. In addition, the mean plant cover in treatment plots was almost twice as high as in control plots.

**Figure 4:**
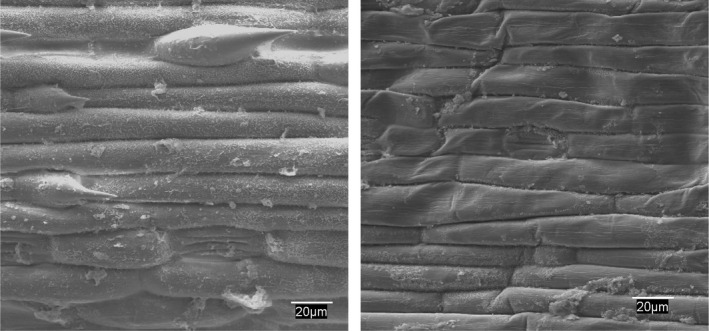
*Bromus matritensis* epidermis. Control with turgid cells and trichomes (left). Collapsed cells 24 hr after treatment (right)

**Figure 5:**
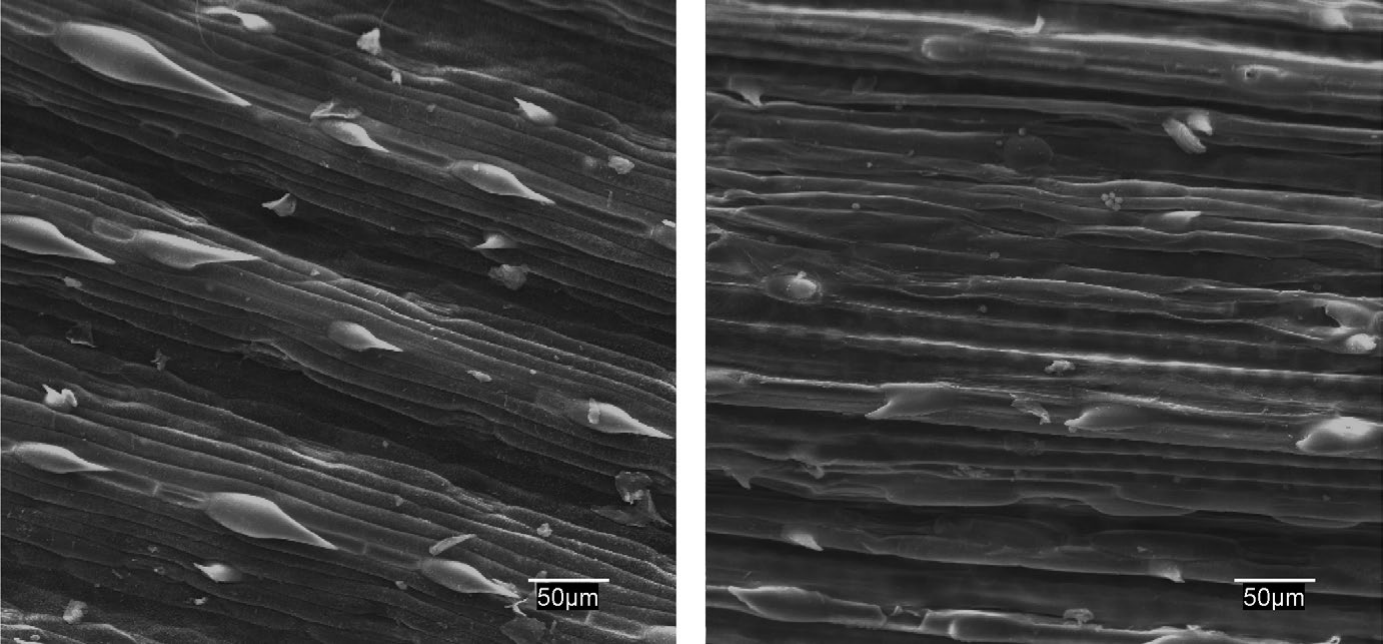
*Hordeum leporinum* epidermis. Control with turgid cells and trichomes (left). Collapsed cells and trichomes 24 hr after treatment (right)

Electron microscopic analysis showed damage to the epidermis and stomatal cells in *Bromus* and *Hordeum* as early as 24 hr after treatment (Figures 4 and 5), with electron micrographs showing damage to the epidermis and loss of cell turgor.

#### 3.3.2 Asteraceae

Asteraceae species are commonly found in nitrophilous communities. Some of the characteristic species include various thistle and sunflower species. In the plots, annual species such as *Carduus pycnocephalus*, *Calendula arvensis*, *Crepis vesicaria,* and *Taraxacum dens‐leonis* were common; spiny biennial species, which spend the first year in rosette and later develop the inflorescence, such as *Eryngium campestre* (Apiaceae) and *Onopordon nervosum* were other common species.

**Figure 6:**
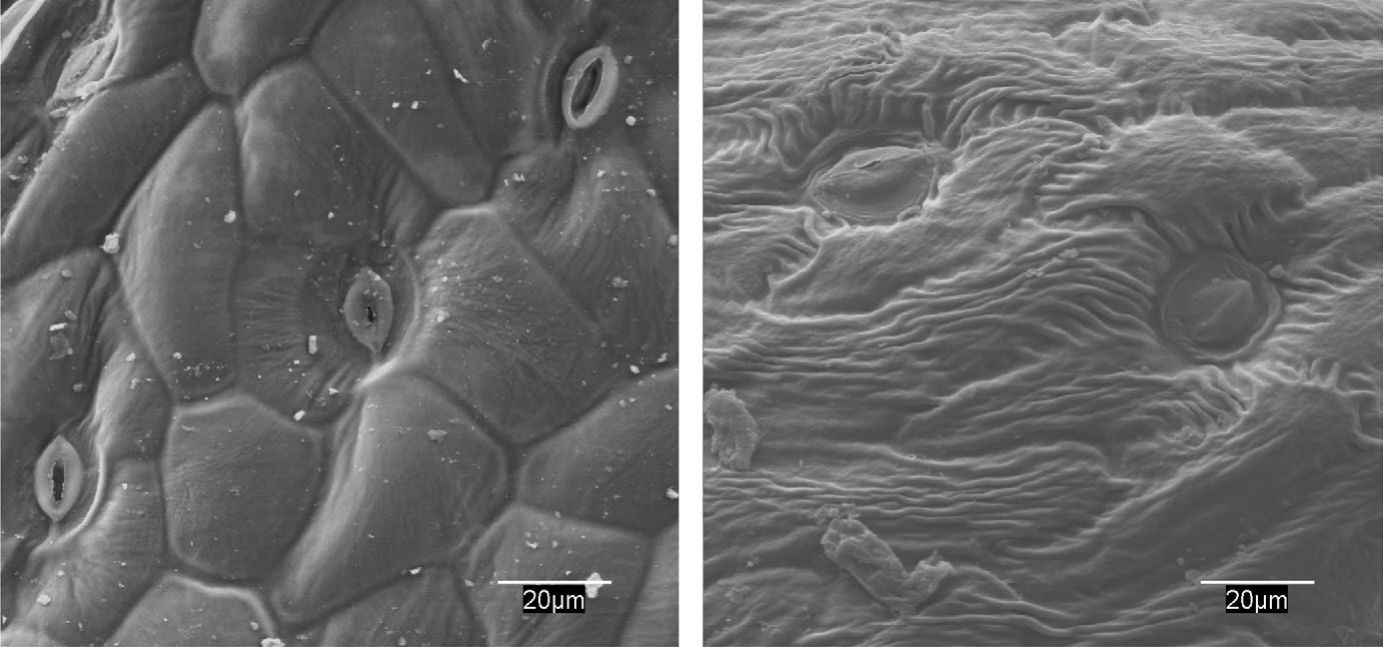
*Carduus pycnocephalus* epidermis. Control with open stomata and turgid cells (left). Closed stomata and large surface wrinkles on cells 24 hr after treatment (right)

**Figure 7:**
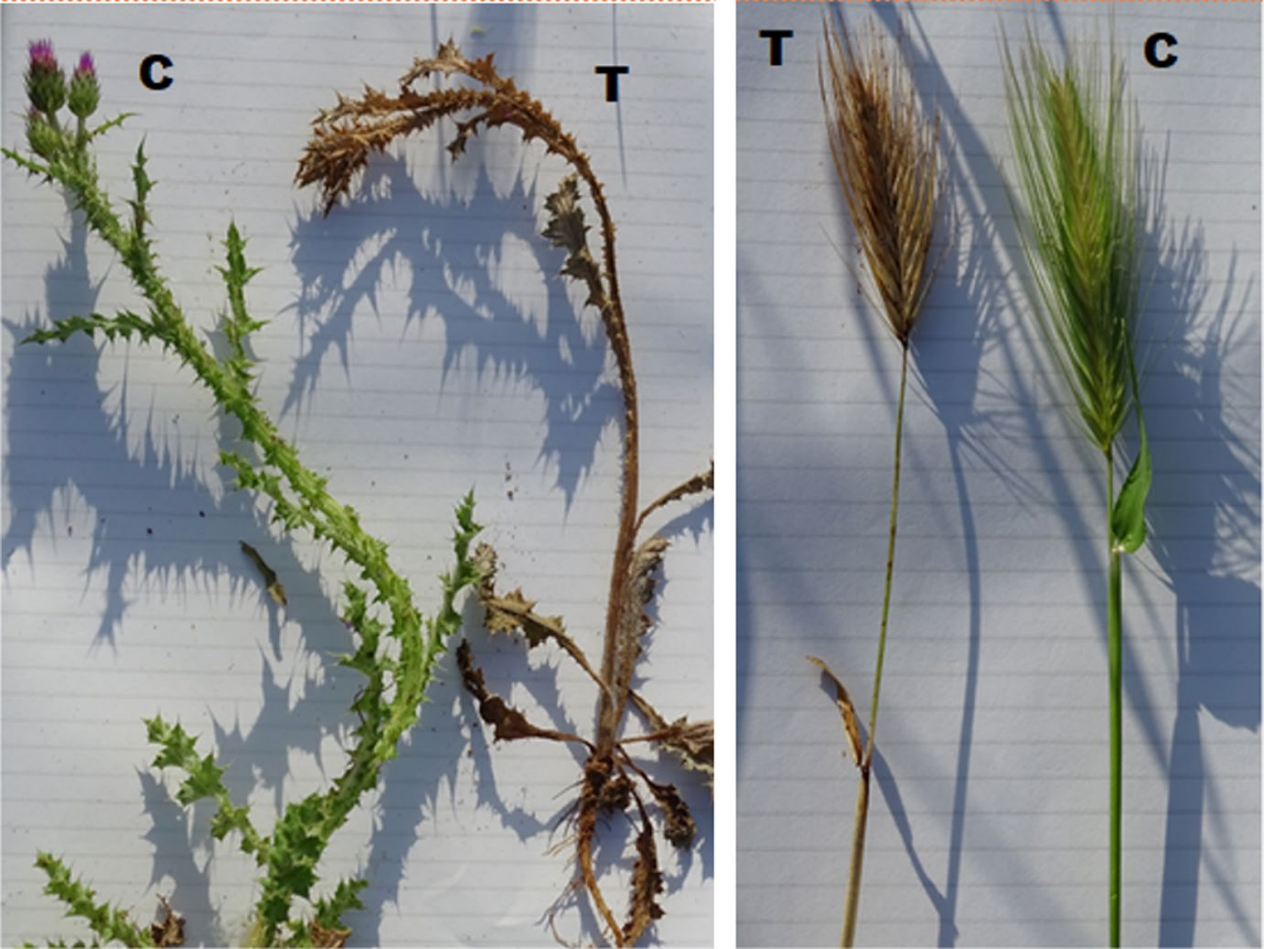
*Carduus pycnocephalus* (left) and *Hordeum leporinum* (right) at 24 hr; control (C), and treatment (T)

WV had a drastic effect on the annual species and eliminated these species within a few hours after its application (Table 9 and Figures 7, 6 and 7). In the case of *Onopordon* and *Eryngium*, WV affected the basal rosette quickly, albeit the root regrew after several weeks. No significant differences were observed between WV treatments, but the differences among the treatments and the control were significant for all dates (ANOVA *p* < .01).

**Table 9:** Percentage of coverage reduction from day 0, for treatments and control

Species	Initial coverage (cm^2^)	Day 7	Day 14	Day 28	Day 42
*Carduus pycnocephalus*	1637.51 ± 672.22	−98.47%	−98.55%	−100%	−100%
Control	1,500 ± 553.55	+25.01%	+125.23%	+126%	+150%
*Onopordon nervosum*	712.5 ± 617.22	−100%	−87.71%	−49.12%	−46.67%
Control	600 ± 70.71	+6.25%	+20.83%	+23.75%	+25%
*Calendula arvensis*	837.5 ± 947.5	−100%	−100%	−100%	−100%
Control	3,000 ± 707.11	0%	+1.66%	+87.91%	+100%

Note

Significant differences were found between the treatments and the control in all intervals and in all species. There were no significant differences between WV treatments (ANOVA *p* > .05).

#### 3.3.3 Geraniaceae

Different species of the *Erodium* and *Geranium* genera are commonly found in nitrophilous communities. All sampled plots had a rather high plant cover of *Geranium molle* and *Erodium cicutarium* as both species are common in all types of nitrate‐rich soils. The WV treatment had an immediate effect on *G. molle*, which practically disappeared in the following samplings (Table 10). The effect on *E. cicutarium* was initially strong, albeit followed by a slow recovery. This was most likely due to its deep roots and thick stems, which allowed these plants to partly recover from the damage caused by WV. Nevertheless, at the end of the study period, their biomass had not reached 50% of the original value, while the corresponding value in the control plot had doubled. When analyzing the results of this species by treatment type, no significant differences were found between WV treatments (ANOVA *p* > .05), but the differences between the treatment and the control plants were significant (ANOVA *p* < .01).

**Table 10:** Percentage of coverage reduction from day 0, for treatments and control

Species	Initial coverage (cm^2^)	Day 7	Day 14	Day 28	Day 42
*Erodium cicutarium*	1,225 ± ± 1,444.19	−84.69%	−63.26%	−50%	−51.02%
Control	750 ± 70.72	+5.02%	+9.00%	+8.33%	+109.01%
*Geranium molle*	2,150 ± 827.3	−100%	−97.67%	−94.76%	−100%
Control	750 ± 35.36	+2.01%	+5.01%	−100%	−100%

Note

Significant differences between treatments and control were found for all intervals in *E. cicutarium* and at 7 and 14 days for *G. molle (*ANOVA *p* < .01*)*. No significant differences were found between WV treatments (ANOVA *p* > .05).

#### 3.3.4 Brassicaceae

The different species of this family, commonly known as mustards, of medium height with white and yellow flowers, dominate a considerable part of nitrophilous communities at their early stages. Genera such as *Diplotaxis*, *Eruca,* and *Sysimbrium* are commonly found in and dominate many ruderal communities. The effect of treatment on *Sysimbrium* was very strong until day 14, at which point the plant started showing some recovery (Table 11). In the control plots, *Sysimbrium* was one of the dominant species in all samplings. The leaves and stems of *Sysimbrium* are thick and wide, which could explain their relative resistance to treatment. When analyzing the results of this species by type of treatment, the differences between the different concentrations of WV were not significant (ANOVA *p* > .05), but they were significant in comparison with the control (ANOVA *p* < .01).

**Table 11:** Percentage of coverage reduction from day 0, for treatments and control

Species	Initial coverage (cm^2^)	Day 7	Day 14	Day 28	Day 42
*Sysimbrium irio*	1,262.5 ± 8,549.63	−87.12%	−74.25%	−26.73%	−88.11%
Control	2,250 ± 70.71	+11.66%	+14.44%	+62.78%	+50%

Note

Significant differences were found between the treatment groups and the control for all intervals *(*ANOVA *p* < .01*)*. No significant differences were found between treatments (ANOVA *p* > .05).

#### 3.3.5 Fabaceae

The main species in the plots was *Medicago sativa*, a perennial herb commonly found in ruderal areas of the Mediterranean region. The effect of treatment on this species was strong at 7 days, with a greater than 85% decrease (Table 12). The species recovered starting at day 14, and at the end of the sampling period (day 42) this species had the second highest plant cover. The different WV treatments showed no significant differences between each other (ANOVA *p* > .05) but significantly differed from the control (ANOVA *p* < .01).

**Table 12:** Percentage of coverage reduction from day 0, for treatments and control

Species	Initial coverage (cm^2^)	Day 7	Day 14	Day 28	Day 42
*Medicago sativa*	1512.5 ± 1,216.02	−85.12%	−39.66%	+28.09%	+46.28%
Control	150 ± 72.20	+25%	+441.66%	+466.6%	+380%

Note

Significant differences between treatments and control were found for all intervals *(*ANOVA *p* < .01*)*. No significant differences were found between treatments (ANOVA *p* > .05).

#### 3.3.6 Perennial versus annual plants

The classification of the set of species in the plots into annual and perennial plants showed significant changes between dates, treatments, and plots. At day 0, when the samplings started, no significant differences were observed in the percentage of annual and perennial plants between different groups (ANOVA perennial *F* = 45, *p* = .65, ANOVA annuals *F* = 0.99, *p* = .41).

After starting the samplings, no significant differences in perennial, biennial, and annual plant cover occurred at any interval in the control plots (ANOVA *p*>.05 in all cases).

In the plots treated with WV, no significant differences occurred between the high, medium, and low treatments in annual or perennial plants on any date (ANOVA *p* > .05 in all cases). Therefore, to analyze the evolution of the treatment plots and to compare them with the control plots, the results were pooled from all plots in which WV was applied.

**Figure 8:**
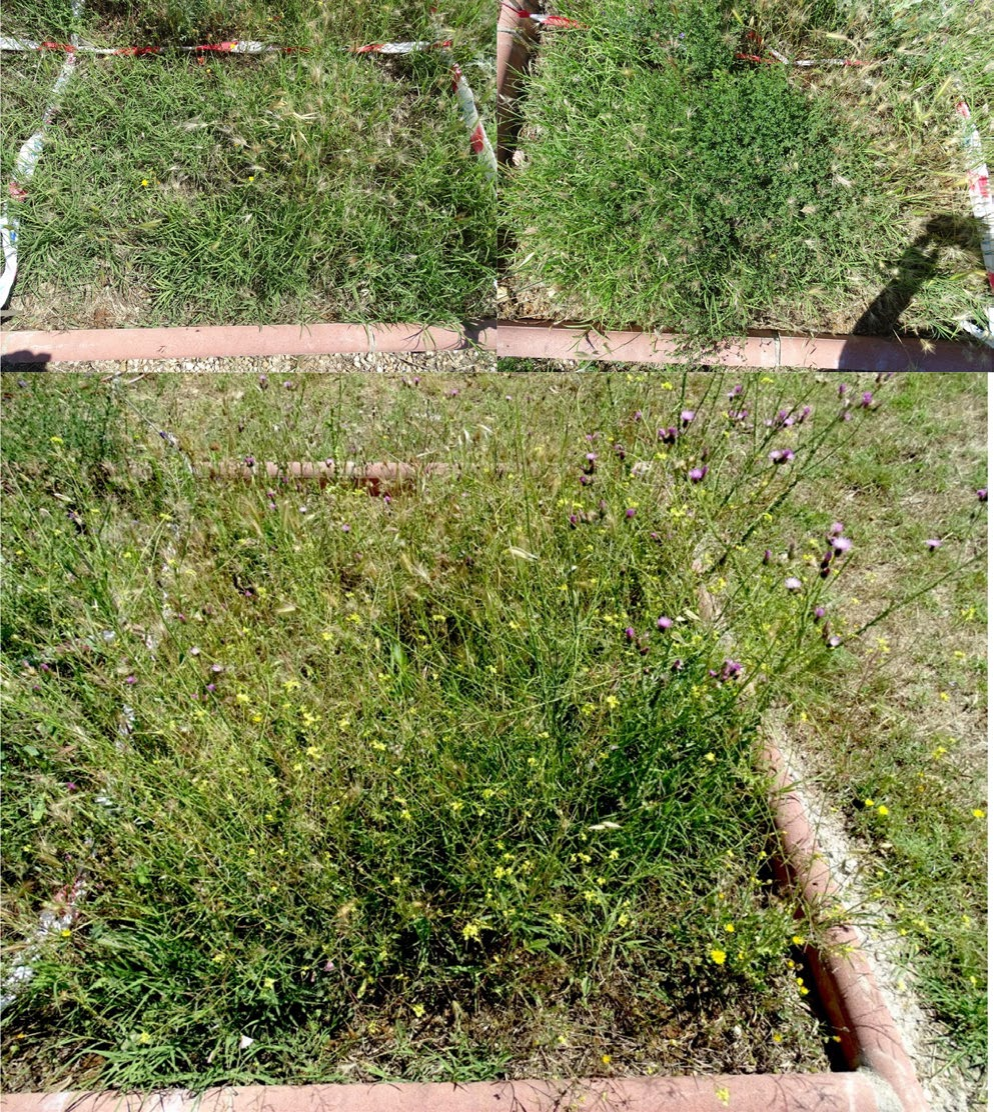
Plots after 42 days, with 100% (above left) and 50% WV (above right). Perennial dominance of *Cynodon dactylon* and *Medicago sativa*. Plot of control with dominance of annuals (*Carduus pycnocephalus* and *Sisymbrium irio*; below)

Over time following WV treatment, the ratio of perennial and annual plants changed significantly (Table 13, Figure 8). In all cases, the plant cover of annual plants was significantly lower at all intervals than at day 0 (ANOVA *F* = 66.91, *p* < .01). However, the plant cover of perennials increased significantly from day 14. (ANOVA *F* = 28.84, *p* < .01, Fisher LSD, 1 vs. 7 *F* = 0.59, *p* = .56; 1 vs. 14 *F* = 1.84, *p* = .07; 1 vs. 28 *F* = 5.64, *p* < .01; 1 vs. 42 *F* = 8.25, *p* < .01; 14 vs. 28 *F* = 3.80, *p* < .01; 28 vs. 42 *F* = 2.61, *p* < .01).

**Table 13:** Occupation percentage of each type of plant in plots treated with wood vinegar (WV) and control plots

Treatment	Initial (cm^2^)	Day 7 (cm^2^)	Day 14 (cm^2^)	Day 28 (cm^2^)	Day 42 (cm^2^)
Perennial (WV)	1725 ± 1,125	1,212 ± 837	3,325 ± 1702	6,637 ± 3,346	8,912 ± 2,594
Control	900 ± 141	975 ± 106	1622 ± 74	1,037 ± 53	3,775 ± 35
Annual (WV)	11,362 ± 2,739	2075 ± 1,230	2,537 ± 1728	2,200 ± 1,429	1,187 ± 925
Control	10,125 ± 1,202	10,597 ± 639	12,082 ± 717	7,607 ± 562	7,567 ± 1,071
Biennial (WV)	750 ± 575	25 ± 81	100 ± 184	425 ± 418	362 ± 608
Control	600 ± 70	637 ± 53	725 ± 35	742 ± 24	750 ± 28

The comparison between the control and treatment plots showed no significant differences in perennial plant cover between days 0 and 14, but the treatment plots had significantly higher perennial plant cover than the control plots from day 14 (Fisher LSD, 1 vs. 14, *F* = 1.87, *p* = .07; 1 vs. 28, *F* = 5.75, *p* < .01; 1 vs. 42, *F* = 8.42, *p* < .01; 14 vs. 28, *F* = 3.88, *p* < .01; 28 vs. 42 *F* = 2.61, *p* < .01).

**Figure 9:**
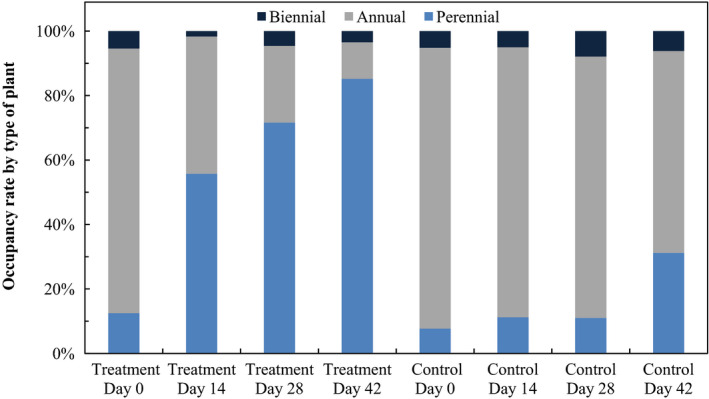
Occupation percentage of each type of plant in plots treated with wood vinegar and control plots

The plant cover of annual plants was higher in control plots than in treatment plots on all dates (ANOVA *F* = 3.35, *p* < .01) (Figure 9).

## 4 DISCUSSION

### 4.1 WV composition by group

**Figure 10:**
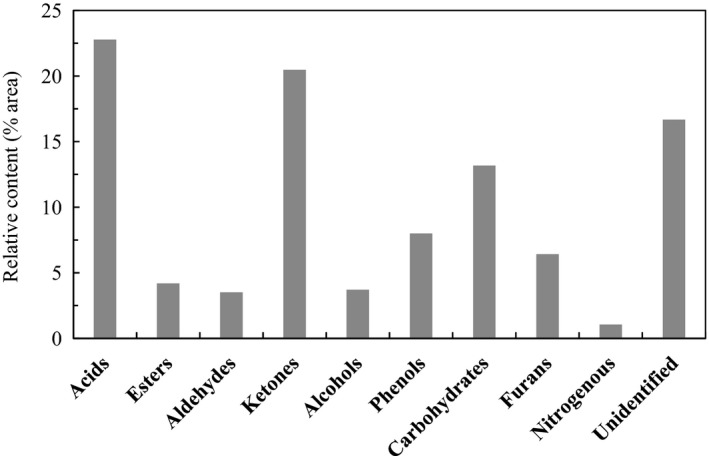
WV composition by compound family

The compounds identified by GC/MS in WV have been classified into carboxylic acids, esters, aldehydes, ketones, alcohols, phenols, carbohydrates, furan derivatives, and nitrogenous compounds (Ma et al., 2014; Souza et al., 2012). Carboxylic acids (22.78% area), ketones (20.48% area), and carbohydrates (13.17% area) were the most abundant groups of compounds in our WV samples. Phenols (8.00% area), furan derivatives (6.43% area), esters (4.20% area), alcohols (3.71% area), and aldehydes (3.52% area) were also identified in significant amounts. Lastly, nitrogenous compounds were also found, albeit in small amounts (Figure 10).

C_2_‐C_4_ monocarboxylic acids derived from thermal decomposition of pine hemicellulose were detected (Cai et al., 2019; Wu et al., 2015; Zhou et al., 2016). Acetic acid was the most abundant compound in WV (18.91% area). Propanoic acid, acetyloxyacetic acid, and butanoic acid were also detected, though in lower proportions (2.30, 1.18, and 0.39% area, respectively). The high carboxylic acid content gave WV a pH value of 2.23, similar to the pH reported in other studies (Aguirre et al., 2020; Fang et al., 2017).

**Figure 11:**
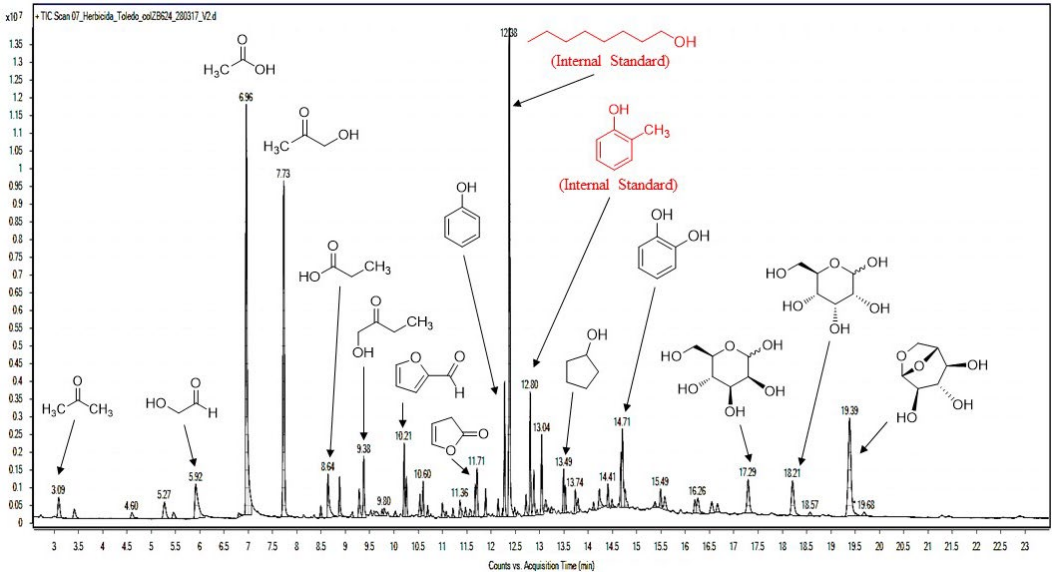
Chromatogram of WV

In addition to carboxylic acids, the thermal decomposition of cellulose and hemicellulose gives rise to a wide variety of low‐molecular‐weight oxygenated organic compounds, such as ketones, aldehydes, alcohols, and furan derivatives (Aguirre et al., 2020; Chen et al., 2019; Demirbaş, 2002; Waters et al., 2017; Zheng et al., 2016), as shown in Figure 11. Among these compounds, 1‐hydroxy‐2‐propanone stands out as the main ketone and the second most abundant compound of WV (13.83% area). Other carbonyl compounds such as hydroxyacetaldehyde (3.29% area), acetone (1.16% area), 2‐cyclopenten‐1‐one (1.18% area), and 2‐hydroxy‐2‐cyclopenten‐1‐one (0.72% area) were identified. Various C_2_‐C_5_ alcohols were also detected, particularly cyclopentanol (1.38% area) and 1,2‐etanodiol (1.31% area).

Three phenols derived from the thermal decomposition of lignin were identified (Lei Chen et al., 2015; A. Demirbaş, 2000; Effendi et al., 2008; Kawamoto, 2017): phenol (3.85% area), catechol (3.24% area), and 2,5‐dimethylphenol (0.91% area). Furfural (2.14% area) was the most abundant furan derivative, followed by 2‐(3*H*)‐furanone (1.77% area) and 2,5‐dihydro‐2,5‐dimethylfuran (0.95% area), among others.

Considerable amounts of several carbohydrates were found. Levoglucosan, a product of cellulose pyrolysis (Fang et al., 2017; Wang et al., 2017), was the most abundant carbohydrate (8.30% area). D‐mannose, a structural monosaccharide of hemicellulose, was also detected (2.27% area). The presence of D‐allose (2.61% area) in the WV is due to the isomerization of sugars derived from hemicellulose during pyrolysis, according to some authors (Wang et al., 2015).

This composition, rich in different organic compounds, provides WV with a wide variety of properties, particularly insecticidal, fungicidal, or herbicidal properties, among others, which have been extensively studied in recent years, (Adfa et al., 2020; Aguirre et al., 2020; Grewal et al., 2018; Ibrahim et al., 2013).

The use of WV as an herbicide has attracted great interest in recent years, due to the search for bio‐herbicidal alternatives to glyphosate. As previously stated, carboxylic acids were the most abundant group of compounds found in the WV, and acetic acid was the main compound. Therefore, acetic acid must be the active component responsible for the herbicidal power of WV, as shown by studies in which acetic acid has been used for the control of some plant species with good results (Benvenuti & Tardivo, 2018). However, acetic acid is not the only compound responsible for the herbicidal properties of WV; the acids, ketones, aldehydes, furans, phenols, and alcohols that make up WV also contribute in different ways and to a greater or lesser extent to this property. Recent studies have demonstrated that WV has much stronger herbicidal properties than pure acetic acid. (Grewal et al., 2018).

### 4.2 Effects on plots

The findings demonstrate that treating plants with WV concentrations ranging from 25% to 100% decreases the biomass development of nitrophilous communities from the first periods of application. No significant differences were found among treatments, which may indicate that concentrations of 25% WV may suffice to control weeds. Moreover, no significant relationship was found between the amount of original biomass and the effect of the treatments, despite the very similar initial biomass amounts across the plots. There are also no differences in the volume used or in the volume‐to‐biomass ratio, which may indicate that the dose of 0.8L/m^2^ suffices to control the herbs at this stage of development in plots.

At 42 days, treatment with 25% WV vol. caused a 15.94% ± 61.32 decrease in biomass, in contrast to decreases of more than 50% in the two other treatments. Despite the nonsignificant results, this treatment with 25% WV vol. had a high *SD* (61.32), which indicated great variability between plots, that is, a much less uniform behavior than that of treatments with higher concentrations of WV (17.48 and 15.04 *SD*). Similar results have been reported in a growth chamber with other species of nitrophilous plants at the same concentration (Aguirre et al., 2020), indicating that a concentration of 25% may not suffice for full weed control with uniform and safe results.

These values contrast with the increase in biomass in the control plots of 282% ± 7.03 at 42 days in comparison with the initial biomass. Therefore, WV clearly has an herb‐control effect and ability to limit biomass development after a single treatment. At 42 days of treatment, the control plots had four‐times more biomass than the treatment plots.

Herb control was also observed in the plant cover data, albeit eventually followed by new surface cover, mainly due to the recovery of some species and to the development of others. The plant cover in the low group at the end of the study period was only 0.44% lower than the initial, with values of 24.63% and 47.64% in the other treatments. The control plots, which already started with rather high plant covers, only partially increased their plant cover after 42 days.

Thus, treatment with WV controlled biomass development in the plot. However, this does not necessarily mean that subsequent occupation of the empty space would not occur. Nitrophilous communities are accustomed to successive waves of germination and covering as the rains continue, until the arrival of the dry season paralyses plant development (Loidi, 2017). However, this recovery is not enough to reach a volume of biomass similar to what the plot would have obtained without the herbicide. In turn, no significant germination of species has been detected in the treatment plots, except for the recovery of some of them and rapid development of others. This could be due to the germination‐inhibiting effect of WV. Even at very low concentrations, WV inhibits germination and seedling development, as previously reported (Luo et al., 2019; Mmojieje & Hornung, 2015). Considering that most annual species in these areas have the potential for long‐term germination over time, this anti‐germination effect of WV would be beneficial for long‐lasting weed control methods.

Our results also showed that some species, such as *Bromus* spp., *Calendula arvensis*, *Carduus pycnocephalus,* and *Geranium molle*, virtually disappeared in a week, with values near 100% reduction. *Sysimbrium irio*, *Hordeum leporinum*, *Onopordon nervosum,* and *Erodium cicutarium* had intermediate values, with plant cover recovery at some intervals. Only two perennial species, *Medicago sativa* and *Cynodon dactylon,* showed increased plant cover by the end of the sampling dates, with significant increases until the last interval. To this point, some species, displaying similar morphological traits, are more sensitive to WV. These annual species grow thin leaves and stems, with few protective structures, and are the main species of ruderal communities. Therefore, the efficacy of this phytosanitary product is promising because the species with thick stems or deep roots, such as *Sysimbrium* or *Erodium* withstand the treatment best, which allows their recovery after treatment, albeit with much lower values than the control.

The electron micrographs at 24 hr of treatment showed considerable deterioration of plant epidermal cells. Therefore, plants with stronger protective structures, such as trichomes or waxes, are likely be more resistant to WV. The mechanism of action of WV on plants is not yet fully clarified, but it seems to involve a combination of the effects of acids and phenols on plant structures and a synergistic effect with the WV. The former would affect vegetative plant parts, and the latter would enable those compounds to stick to the plant, thereby promoting their action. For example, phenols affect germination by reducing amylase activity in weeds, which delays seed germination due to the slow starch‐hydrolysis process (Radhakrishnan et al., 2018). Seedling leaves also show drastic degenerative changes with other phenolic compounds (Tigre et al., 2015). Furthermore, the effect of fatty acids on plants has physiological implications, which could be linked to the increased physical activity of acids in tissues (Aguirre et al., 2020; Fukuda et al., 2004).

One of the objectives of the present study was to identify the type of plant succession that occurred after applying WV. Under controlled conditions, the natural processes of competition, germination, and dispersion are difficult to reproduce. Accordingly, it is important to determine the plant processes that can occur in a plot after this type of treatment.

The results indicate that after the species most sensitive to WV disappear, other species colonize the plot; however, this recovery is not enough to reach biomass levels similar to those that would have been reached without applying WV. In turn, *Erodium* and *Sysimbrium* species grow, and these species may have tissue reserves for a second growth, but colonization with perennial species is the prevailing outcome. Perennial species usually have a later phenology, so a treatment in early spring has little effect on them. Their endurance then allows perennials to fill the space left by the annuals reduced by the treatment.

Despite starting with a very similar annual/perennial composition among plots on day 0, at the end of the sampling period the perennial species accounted for more than 80% of the plant cover in the treatment plots, in contrast to 30% in the control plots. Therefore, these species benefit from the elimination of competition with annual plants. Even still, such perennial species do not reach their optimum in these ruderal ecosystems (Loidi, 2017; Rendeková et al., 2018); although their plant cover is higher than expected, the biomass that they reach at the end of the sampling period is much lower than that of annual species grown in control plots.

The efficacy of this phytosanitary product depends on the objective of the herbicidal treatment. When the desired outcome is to limit biomass development, especially due to the effects of competition on crops, the phytosanitary product is effective if it reduces biomass development and therefore competition for resources with the crops. In addition, by delaying weed germination and growth over time, it can help crop development because this factor (sprouting time) is one of the key factors that controls competition between wild plants and crops (Christoffoleti et al., 2007).

When the objectives are more esthetic, for example, using herbicides in gardens or urban spaces, replacing annual with perennial species can be very positive in the medium term. A way to avoid the successive emergence of nitrophilous communities is to occupy the space with other types of species (Upadhyaya & Blackshaw, 2007). If the treatment with WV favors the emergence of perennial species, this can change the dynamics of the environment because the perennial plant cover, by preventing light from reaching the ground, reduces or prevents the emergence of many annual species (Jensen, 1995). In any case, these findings must be corroborated with new experiments in the field.

When the objective of the herbicide treatment is to completely remove the plant cover in an area, WV is inadequate, especially with a single treatment. As a contact and nonsystemic herbicide, WV does not prevent some plant dynamics in the environment. This partial effect may preclude some of its possible applications; however, this is advantageous from an environmental standpoint, because WV does not produce an area devoid of vegetation, unlike other types of treatments, instead reducing growth and changing the composition of the plot. Another option would be to perform several treatments in one area, thereby controlling the development of all species, including perennials, but this approach may not be economically feasible.

Another variable that may reduce the effectiveness of the phytosanitary product is the dilution effect that may occur in the area when it rains. Given the speed of action of the phytosanitary product, which reaches peak efficiency after 7 days, WV should be applied in the dry season to prevent rainfall from diluting the phytosanitary product.

An increasing number of studies have examined the possible environmental impact on the soil from using WV. Many studies have recently assessed the effect of the herbicide on the soil microbiota and its main effects on soil or aquatic organisms (Koç et al., 2019; Steiner et al., 2008). Most concluded that WV improves soils conditions, favors the microbiota, and somewhat invigorates soil organisms. Maliang et al. (2020) studied the effect of continuous WV application on the soil pH. After monitoring the pH value for 18 days, they demonstrated that, despite the decrease in pH on the third day of application, the use of WV had no long‐term effects, and the original pH value was gradually restored. WV has also been used as a fertilizer in low concentrations. Some articles have found a somewhat negative effect on aquatic organisms, which, if confirmed, should lead to a series of regulations on its use in areas near streams or surface waters (de Lima et al., 2019).

One of the most problematic aspects of using WV as an herbicide is obtaining legal authorization for its use as an herbicide or as a phytosanitary product. Most laws on herbicide products worldwide, and especially in European legislation, are developed to certify the uses of specific substances or chemical compounds (i.e., the action of a single substance or molecule). Some product formulations that are based on an authorized substance add a known sum of other products in the form of additives. However, WV is a mixture of more than 200 specific compounds, which may hinder the acquisition of necessary permits for its use as an herbicide, for example, based on current European legislation, particularly Regulation (EC) No 1907/2006 on the Registration, Evaluation, Authorisation, and Restriction of Chemicals (REACH). This regulation contains an authorization category of mixtures under the category of multiconstituent substances or substances of Unknown or Variable composition, Complex reaction products, or Biological materials (UVCB substances), which apply to substances with a large number of constituents in variable amounts, often little known. This category would be used for WV.

From the standpoint of organic farming legislation, regulated at the European level by the Regulation (EU) 2018/848 of the European Parliament and of the Council of 30 May 2018 on organic production and labeling of organic products, the use of WV would fall under article 5, of general principles of organic production, which details the use of natural substances or derivatives of natural substances. This provision offers a possible path for the authorization of WV if classified as a wood derivative. Vinegar has been used as an herbicide in recent years. This product has been authorized by including vinegar as a basic substance based on the Commission Implementing Regulation (EU) 2015/1108 of 8 July 2015 approving the basic substance vinegar. This declaration of vinegar as basic substance No. CAS: 90132‐02‐8 of 1 July 2015 has enabled the use of vinegar as a fungicide and bactericide for organic farming. Previously, acetic acid had been included based on Regulation 2008/127/CE of 1/09/2009 as an active substance with an herbicidal function in the European Union lists of approved, nonapproved, and low‐risk active substances, substances under review, and basic substances. This could be the path toward selling WV in the European Union and in other countries with similar legislation.

## 5 CONCLUSIONS

WV is a complex mixture with numerous components, predominantly acids, ketones, phenols, and carbohydrates. Treatment with WV helps to control the development of annual plants in ruderal communities. This control decreases the growth of some species and eliminates others. After applying WV, annual plants are replaced by perennial plants, which may be important in some treatments. The WV effect is detected within a few hours after is application, affecting the entire epidermis of the plant and its stomatal cells. The most resistant species have thicker stem and leaf structures or substances from underground reserves. In conclusion, WV is a useful compound for some herb‐control treatments, effectively controlling biomass development, and replacing systemic herbicides with WV may be important in some cases. In order to sell WV as an herbicide, some legal obstacles related to its complex composition must be overcome.

## CONFLICTS OF INTEREST

The authors declare no conflict of interest.
